# Supplementary material for: The Impact of a Structured Outpatient Parenteral Antimicrobial Therapy (OPAT) Programme on Quality of Care, Optimisation of Antimicrobial Use, and Healthcare Costs: A Retrospective Cohort Study
Source: Antibiotics (Basel). 2025 Nov 2;14(11):1103. doi: 10.3390/antibiotics14111103 (PMC12649236; doi:10.3390/antibiotics14111103)
Supplement: Supplementary file 1 [file antibiotics-14-01103-s001.zip › antibiotics-3935781-supplementary.pdf]

Supplementary Materials

Table of contents

Page 2: Table S1: Prices per day of outpatient IV antimicrobials per year.
Page 2: Table S2: Prices per day of oral antimicrobials (2025).
Page 3: Table S3: Costs for PICC placement, inpatient days, OPAT team, and TDM.
Page 3: Table S4: Number of days (%) of IV antimicrobials prescribed, per year.
Page 4: Table S5: Number of days of oral antimicrobial alternatives prescribed in the OPAT cohort, per year.

Table S1: Prices per day of outpatient IV antimicrobials per year.

	Dosage	2019	2022	2023	2024
Acyclovir	30 mg/kg	€ 170,00	€ 170,00	€ 170,00	€ 170,00
Benzylpenicillin	6, 12 or 18 million IU/24h	€ 176,75	€ 207,22	€ 223,80	€ 233,64
Flucloxacillin	6 g/24h	€ 158,69	€ 190,51	€ 205,75	€ 214,80
Flucloxacillin	12 g/24h	€ 171,12	€ 203,73	€ 220,03	€ 229,71
Amoxicillin	6 or 12 g/24h	€ 170,00	€ 170,00	€ 170,00	€ 170,00
Cefazoline	3, 4 of 6 g/24h	€ 170,00	€ 170,00	€ 170,00	€ 170,00
Cefuroxime	2250 or 4500 mg/24h	€ 132,10	€ 138,32	€ 149,39	€ 155,96
Ceftriaxone	2 or 4 g/24h	€ 170,00	€ 129,95	€ 140,34	€ 146,52
Cefotaxime	2 or 4g/24h	€ 170,00	€ 170,00	€ 170,00	€ 170,00
Ceftazidime	1 or 1.5g/24h	€ 170,00	€ 108,61	€ 117,30	€ 122,46
Ceftazidime	3 g/24h	€ 170,00	€ 119,28	€ 128,82	€ 134,49
Ceftazidime	4 or 4.5g/24h	€ 170,00	€ 124,59	€ 134,56	€ 140,48
Ceftazidime	6 g/24h	€ 170,00	€ 140,46	€ 151,70	€ 158,37
Meropenem	3 g/24h	€ 170,00	€ 150,33	€ 162,63	€ 169,50
Ertapenem	1000mg/24h	€ 170,00	€ 170,00	€ 170,00	€ 170,00
Vancomycin	2500 mg	€ 170,00	€ 140,22	€ 140,22	€ 140,22
Piperacillin/tazobactam	12/1.5g/24h	€ 170,00	€ 170,00	€ 47,15	€ 47,15
Ciprofloxacin	1200 mg/24h	€ 170,00	€ 170,00	€ 170,00	€ 170,00
Gentamicin	3 or 5 mg/kg	€ 170,00	€ 170,00	€ 170,00	€ 170,00
Liposomal Amphotericin B	4 mg/kg	€ 170,00	€ 170,00	€ 170,00	€ 170,00
Anidulafungin	100 mg	€ 170,00	€ 170,00	€ 170,00	€ 170,00
Caspofungin	70 mg	€ 170,00	€ 170,00	€ 170,00	€ 170,00

Table S2: Prices per day of oral antimicrobials (2025).

	Dosage	Price per day
Amoxicillin	3dd 750mg	€ 0,72
Amoxicillin-clavulanate	4dd 500/125mg	€ 1,68
Azithromycin	1dd 500mg	€ 0,89
Cefpodoxime	4dd 500mg	€ 2,12
Cefuroxime	2dd 500mg	€ 1,64
Ciprofloxacin	2dd 750mg	€ 0,76
Clindamycin	3dd 600mg	€ 3,72
Trimethoprim-sulfamethoxazole	2dd 960mg	€ 0,52
Doxycycline	2dd 100mg	€ 1,26
Flucloxacillin	4dd 500mg	€ 1,16
Fluconazole	1dd 200mg	€ 0,47
Fosfomycin	1dd 3g	€ 2,49
Levofloxacin	2dd 250mg	€ 1,68
Linezolid	2dd 600mg	€ 23,24
Metronidazole	3dd 500mg	€ 0,93
Nitrofurantoin	2dd 100mg	€ 0,62
Pivmecillinam	3dd 400mg	€ 5,85
Rifampicin	2dd 450mg	€ 2,64
Valganciclovir	1dd 450mg	€ 4,27
Voriconazole	2dd 200mg	€ 3,34

Table S3: Costs for PICC placement, inpatient days, OPAT team, and TDM.

	2019	2022	2023	2024
PICC placement	€ 225,00	€ 225,00	€225,00	€ 225,00
Inpatient day[1]	€ 525,00	€ 550,00	€ 725,00	€ 725,00
OPAT team[2]	€ 23,43	€ 23,43	€ 23,43	€ 23,43
TDM	€ 6,99	€ 6,99	€ 6,99	€ 6,99

[1]The average daily cost of admission to the internal medicine department.
[2]Costs of the OPAT team for 15 minutes per patient.

Table S4: Number of days (%) of IV antimicrobials prescribed, per year.

	Dosage	2019	2022 (15th Aug – 31st Dec)	2023	2024	p-value *
Acyclovir	30 mg/kg	7 (0.2%)	0	18 (1.2%)	7 (0.3%)	0.0319
Benzylpenicillin	6, 12 or 18 million IU/24h	264 (8.8%)	130 (18.4%)	291 (19.7%)	510 (25.3%)	< 0.001
Flucloxacillin	6 g/24h	298 (9.9%)	55 (7.8%)	88 (5.9%)	82 (4.1%)	< 0.001
Flucloxacillin	12 g/24h	158 (5.2%)	160 (22.7%)	227 (15.3%)	140 (6.9%)	< 0.001
Amoxicillin	6 or 12 g/24h	7 (0.2%)	0	54 (3.7%)	150 (7.4%)	< 0.001
Cefazoline	3, 4 or 6 g/24h	44 (1.5%)	0	23 (1.6%)	44 (2.2%)	0.1475
Cefuroxime	2250 or 4500 mg/24h	211 (7%)	0	0	55 (2.7%)	< 0.001
Ceftriaxone	2 or 4 g/24h	413 (13.7%)	13 (1.8%)	204 (13.8%)	353 (17.5%)	0.0177
Cefotaxime	2 or 4g/24h	334 (11.1%)	0	0	0	< 0.001
Ceftazidime	1 or 1.5g/24h	35 (1.2%)	19 (2.7%)	0	12 (0.6%)	0.0062
Ceftazidime	3 g/24h	14 (0.5%)	10 (1.4%)	0	0	0.0049
Ceftazidime	4 or 4.5g/24h	10 (0.3%)	0	0	6 (0.3%)	0.2476
Ceftazidime	6 g/24h	90 (3.0%)	173 (24.5%)	119 (8.0%)	107 (5.3%)	< 0.001
Meropenem	3 g/24h	801 (26.5%)	84 (11.9%)	175 (11.8%)	121 (6.0%)	< 0.001
Ertapenem	1000mg/24h	0	33 (4.7%)	0	0	0.2004
Vancomycin	2500 mg	280 (9.3%)	17 (2.4%)	214 (14.5%)	356 (17.6%)	< 0.001
Piperacillin/tazobactam	12/1.5g/24h	0	11 (1.6%)	15 (1%)	54 (2.7%)	< 0.001
Ciprofloxacin	1200 mg/24h	0	0	22 (1.5%)	0	0.0015
Gentamicin	3 or 5 mg/kg	12 (0.4%)	0	0	22 (1.1%)	0.0543
Amphotericin B	4 mg/kg	0	0	7 (0.5%)	0	0.0732
Anidulafungin	100 mg	39 (1.3%)	0	0	0	<0.001
Caspofungin	70 mg	0	0	22 (1.5%)	0	0.0015
**Total**		**3017 (100%)**	**705 (100%)**	**1479 (100%)**	**2019 (100%)**	

*= Cochran-Armitage test for trend

Table S5: Number of days of oral antimicrobial alternatives prescribed in the OPAT cohort, per year.

	Dosage	2022 (15th Aug – 31st Dec)	2023	2024
Amoxicillin	3dd 750mg	0	57	36
Amoxicillin-clavulanate	4dd 500mg	48	83	17
Azithromycin	1dd 500mg	0	0	8
Cefpodoxime	4dd 500mg	0	0	14
Cefuroxime	2dd 500mg	48	3	0
Ciprofloxacin	2dd 750mg	95	205	288
Clindamycin	3dd 600mg	366	356	272
Trimethoprim-sulfamethoxazole	2dd 960mg	0	6	42
Doxycycline	2dd 100mg	36	0	0
Flucloxacillin	4dd 500mg	0	8	0
Fluconazole	1dd 200mg	0	18	0
Fosfomycin	1dd 3g	22	20	10
Levofloxacin	2dd 250mg	31	381	160
Linezolid	2dd 600mg	4	95	13
Metronidazole	3dd 500mg	0	62	28
Nitrofurantoin	2dd 100mg	0	15	34
Pivmecillinam	3dd 400mg	0	65	0
Rifampicin	2dd 450mg	103	232	150
Valganciclovir	1dd 450mg	0	0	7
Voriconazole	2dd 200mg	0	19	0
